# Breaking the Data Value-Privacy Paradox in Mobile Mental Health Systems Through User-Centered Privacy Protection: A Web-Based Survey Study

**DOI:** 10.2196/31633

**Published:** 2021-12-24

**Authors:** Dongsong Zhang, Jaewan Lim, Lina Zhou, Alicia A Dahl

**Affiliations:** 1 The University of North Carolina at Charlotte Charlotte, NC United States

**Keywords:** mobile apps, mental health, privacy concerns, privacy protection, mobile phone

## Abstract

**Background:**

Mobile mental health systems (MMHS) have been increasingly developed and deployed in support of monitoring, management, and intervention with regard to patients with mental disorders. However, many of these systems rely on patient data collected by smartphones or other wearable devices to infer patients’ mental status, which raises privacy concerns. Such a value-privacy paradox poses significant challenges to patients’ adoption and use of MMHS; yet, there has been limited understanding of it.

**Objective:**

To address the significant literature gap, this research aims to investigate both the antecedents of patients’ privacy concerns and the effects of privacy concerns on their continuous usage intention with regard to MMHS.

**Methods:**

Using a web-based survey, this research collected data from 170 participants with MMHS experience recruited from online mental health communities and a university community. The data analyses used both repeated analysis of variance and partial least squares regression.

**Results:**

The results showed that data type (*P*=.003), data stage (*P*<.001), privacy victimization experience (*P*=.01), and privacy awareness (*P*=.08) have positive effects on privacy concerns. Specifically, users report higher privacy concerns for social interaction data (*P*=.007) and self-reported data (*P*=.001) than for biometrics data; privacy concerns are higher for data transmission (*P*=.01) and data sharing (*P<*.001) than for data collection. Our results also reveal that privacy concerns have an effect on attitude toward privacy protection (*P*=.001), which in turn affects continuous usage intention with regard to MMHS.

**Conclusions:**

This study contributes to the literature by deepening our understanding of the data value-privacy paradox in MMHS research. The findings offer practical guidelines for breaking the paradox through the design of user-centered and privacy-preserving MMHS.

## Introduction

### Patient Data Privacy

Mental health, including emotional, psychological, and social well-being, affects how people think, feel, and act. According to the National Alliance on Mental Illness, in the United States, 1 in 5 adults experience a mental illness; depression, a type of mental disorder, is the leading cause of disability worldwide; and 90% of the people who commit suicide have mental illness. Recent trends in the health care industry have been driving significant changes in the health information technology landscape, including the movement toward developing effective technologies that enable continuous data collection from patients through mobile and wearable devices [1]. Examples of these trends include the shift of health care systems toward more efficient yet less expensive methods of patient care; strong economic incentives to pursue continuous patient monitoring outside clinical settings and innovative technologies to prevent patients from falling ill; increasing adoption of mobile and wearable devices such as smartphones and biological sensors by patients, caregivers, and health care service providers for health and wellness apps; and technology advances that increase the utility of mobile devices [1].

Rapid advances in wireless communication, low-power sensing technologies, and pervasive mobile and wearable devices (eg, smartphones, smart watches, and Fitbit) propel research on, and practice of, mobile health (mHealth), including mobile mental health (MMH). According to the Pew Research Center [2], 81% of American adults have a smartphone. More than 60% of people have downloaded an mHealth app, with more than 300,000 mHealth apps available. The main features of mHealth apps include symptom checkers, health care professional finders, management of clinical records, medical education and training, patient monitoring, patient self-management, and prescription filling and compliance [3].

MMH systems (MMHS) collect unprecedented amounts and varieties of data through sensors, smartphones, or other wearable devices in support of continuous monitoring, self-management, and intervention with regard to patients with mental illness or patients’ well-being. These data enable researchers to quantify complex temporal dynamics of important physical (eg, body movement), biological (eg, skin temperature and heart rate), behavioral (eg, phone use behavior and keystrokes), psychological (eg, emotion), social (eg, social interactions with others such as phone calls and SMS text messaging), and environmental factors (eg, location and lighting) that may be affected by, or be indicative of, mental illness [4-6]. Thus, MMH technology has great potential to yield new insights, increase health care agility and quality, extend ubiquitous access to health care resources and services, reduce hospital admissions and cost, and improve personal wellness and public mental health.

These benefits, however, can only be achieved if the health-related data continuously collected from individuals by MMHS are appropriately protected for user privacy. The general notion of privacy is perceived as a human right, a commodity, and control [7]. This research focuses on patient data privacy during collection, transmission, storage, and sharing of personal data. Unlike data security, which refers to physical, technological, or administrative safeguards or tools used to protect identifiable health data from unwarranted access or disclosure [8], health information privacy is an individual’s right to control the acquisition, use, or disclosure of their identifiable health-related data, including when, how, and to what extent the data can be communicated to others [9]. Vulnerabilities regarding privacy may result in breaching the conﬁdentiality of patient data [10], leading to ﬁnancial losses, discrimination, stress, dissatisfaction, or even delays in seeking timely treatment because of perceived privacy risks. Individuals with high privacy concerns often perceive a new information system to be risky, eventually developing concerns about it [10].

Despite its potential, mHealth research and practice has progressed much more slowly than app developments in the industry because privacy issues remain an ongoing concern because of the sensitive, personal, and streaming nature of data collected from individual patients by sensors or other wearable devices [11]. Our literature review reveals that approximately half of the surveyed studies on MMHS [12-14] did not consider data privacy issues at all. Prior research also suggests that users lack understanding of privacy issues associated with mHealth technologies [9]. Although some studies adopted certain user privacy protection methods, most of them deployed a single method (eg, data encryption [15-17] and extracting and storing features of data instead of original content [18,19]). A number of studies have shown that users sometimes sacrifice their privacy in exchange for benefits and personalized services [20,21]. Different types of information may have different levels of overall “privateness [22].” There is a severe lack of studies and comprehensive understanding of users’ privacy concerns with different types of personal data collected and used by MMHS and how to address them to increase users’ adoption of, and engagement with, these systems [23].

According to the privacy calculus theory [24], an individual’s intention to disclose personal information is based on their perceived risk and anticipated benefits. On the one hand, it is theoretically desirable for MMHS to collect as much (and detailed) relevant personal data as possible from individuals that are indicators of mental health so that the systems can predict the individuals’ mental status more accurately and make more informed intervention decisions. On the other hand, it remains uncertain how sensitive, in terms of privacy, users are to different types of personal data being collected, which data processing stages may cause them to have privacy concerns, and to what extent privacy concerns may influence their willingness to use MMHS. To help address the data value-privacy paradox, this study aims to answer the following research questions:

- Research question 1: How do users’ privacy concerns vary with different types of personal data collected by MMHS?
- Research question 2: Do users’ privacy concerns vary with different data processing stages that MMHS involve? If so, how?
- Research question 3: How do privacy concerns affect users’ intention of using MMHS?

To answer these research questions, we conducted a web-based survey with adults who have self-reported mental health issues and used MMHS before. On the basis of the findings of our survey, we propose a set of guidelines for the design of user-centric and privacy-protecting MMHS. This study contributes to MMHS research by deepening our understanding of users’ privacy concerns and potential mitigation solutions. In addition, it offers practical implications for improving the well-being of patients with mental illness by cultivating their adoption of, and engagement with, MMHS.

### Background and Related Work

#### Conceptualization of Privacy

Generally, privacy can be categorized into physical privacy and information privacy (also commonly referred to as data privacy). Historically, the concept of physical privacy was defined as “the right to be left alone [25].” Information privacy is concerned not only with individuals’ personal information such as name, home address, and birth date, but also their relationship status, photographs, political and religious views, shopping habits, driving history, and medical records [26]. It also involves an individual’s ability to control information about themselves [27]. Information privacy is also referred to as controlling whether and how personal data can be collected, stored, processed, and disseminated [28]. As technologies evolve, privacy has been increasingly threatened as a result of the rapid growth of portable handheld devices, sensors, and wireless network technology. Accordingly, the conceptualizations of privacy have shifted toward elaborating the complexity of privacy issues in various areas involving the legal, social-psychological, economic, or political concerns that technologies present.

Smith et al [7] proposed a macro model called “Antecedents→Privacy Concerns→Outcomes” that demonstrated the relationships between privacy concerns and their antecedents and outcomes. The model shows that individuals’ experiences with getting exposed to, or victimized by, personal information abuses; privacy awareness; personality (eg, introversion vs extroversion); demographics; and cross-cultural differences are antecedents of privacy concerns. Privacy concerns in turn affect behavioral reactions (eg, willingness to disclose information), trust, regulation, and privacy calculus (ie, trade-off between privacy risks and benefits).

Plachkinova et al [29] developed a taxonomy based on security challenges in an mHealth care environment defined by Stavrou and Pitsillides [30] and the threat taxonomy for mHealth privacy proposed by Kotz [31]. Plachkinova et al [29] identified a few common threats to privacy, including (1) identity threats: misuse of patient identity information; (2) access threats: unauthorized access to protected health information (PHI) or personal health records; and (3) disclosure threats: unauthorized disclosure of patient identity information or PHI. However, the authors’ taxonomy neither differentiates data types nor considers user privacy protection.

#### Privacy Regulations

Privacy regulations have been established to help determine effective ways to develop, manage, monitor, and enforce patient-centric, organizational, and government policies and regulations associated with data collection and use within mHealth systems [32]. For example, the Health Insurance Portability and Accountability Act (HIPAA) of 1996 provides data privacy and security guidelines for safeguarding medical information and sets constraints and conditions for the use and disclosure of patient information (Textbox 1). HIPAA’s privacy rule only applies to mHealth apps that involve both a covered entity (eg, health care providers) and PHI. PHI usually includes demographic information, medical history, diagnostic test results, insurance information, and other data gathered by a health care professional that identify an individual and are used for medical treatment. HIPAA does not cover individual users who upload or directly enter their information into mHealth apps [33]. In addition, researchers must abide by the federal policy for the protection of human subjects, also known as the Common Rule, to protect individuals participating in research activities. The Common Rule specifies detailed policies and guidelines about informed consent, adverse events, handling of biological data, and vulnerable populations, among other issues [34].

**The Health Insurance Portability and Accountability Act privacy and security requirements (adapted from Ray and Biswas [35]).**

**Privacy and security requirements**

- Patients’ understanding
- Patients have the right to understand how their health information will be used and stored.
- Patient control
- Patients can control the access to their health information and are given permission to decide who can access their health data.
- Confidentiality
- Health data of patients must be kept undisclosed from any party that has no right to access the data.
- There should be software safeguards such as encryption to protect health data confidentiality during storage and transmission.
- Data integrity
- Patients’ eHealth information should be protected from omissions, tampering, and unauthorized destruction.
- The health data shared with an entity must be the true representation of the intended information without having any form of alteration.
- Consent exception
- In life-saving purposes and emergency situations, access to the protected health information without the patient’s authorization is allowed.

The most recent US privacy regulation is the California Consumer Privacy Act, which provides California residents transparency and protection of personal data, including the right to know where their data are collected and to whom they are sold, as well as the right to disclose. In 2019, Xcertia [36] published the following industry guidelines for safe and effective mHealth apps:

- Guideline P1: Notice of Use and Disclosure. The Privacy Notice describes how an organization collects, uses, and retains user data.
- Guideline P2: Retention. If data are collected, the user shall be informed about how long the data will be retained.
- Guideline P3: Access Mechanisms. An app user should be informed if the app accesses local resources or resources from, or for, social networking platforms, provided with an explanation by any appropriate means (eg, the About section) as to how and why such resources are used, and opt-in consent should be obtained to access such resources.
- Guideline P4: HIPAA Entity or Business Associate. If a mobile app collects, stores, or transmits information that constitutes PHI (as defined by HIPAA), it does so in full compliance with HIPAA and all applicable state and international regulations.
- Guideline P5: Children’s Online Privacy Protection Act. An app should have measures in place to protect children in accordance with applicable laws and regulations if the website is directed at children.
- Guideline P6: General Data Protection Regulation. An app should have measures in place to comply with applicable laws and regulations related to the European Union General Data Protection Regulation.

#### Personal Data Collected by Existing MMH Studies

To understand what personal data have been collected by existing MMH studies and whether these studies have deployed any privacy protection method, we first conducted a literature review. We formulated search queries as various combinations of terms from 3 groups, including technology terms such as “mobile,” “wearable devices,” “sensor,” “IoT,” and “mobile app;” mental health terms such as “mental health,” “depression,” “schizophrenia,” and “stress management” used by Bardram and Matic [37] and the US Department of Health and Human Services; and privacy-related search terms such as “privacy,” “privacy protection,” “personal,” and “private information.” We searched for relevant articles in the following databases: PubMed, IEEE Xplore, National Institute of Mental Health Data Archive, ScienceDirect, Taylor & Francis Online, and PubPsych, as well as Google Scholar. We applied 3 inclusion criteria in paper selection: (1) published in English in or after 2014 to reflect the state of the art, (2) focused on MMHS in support of users with an existing mental disorder, and (3) collected personal data from users.

We found and reviewed 32 papers that met the aforementioned inclusion criteria. These studies [4-6,12-19,38-58] are summarized in Multimedia Appendix 1 along the following dimensions: the target mental diseases that the MMHS were proposed to support, types of personal data collected by the apps, and privacy protection methods deployed in these studies, if any. These studies collected a wide variety of personal data from patients, driven by the target mental diseases. The most commonly used personal data are as follows:

- Physical activities such as gait, finger tapping, activity time, and distance traveled
- Sleep data such as sleeping time and waking time
- Physiological data (biomarkers) such as oxygen saturation, heart rate, temperature, blood pressure, electrocardiogram, and peak expiratory flow rate
- Location and GPS data
- Social activity (ie, social interaction) data
- Phone use such as number and length of phone calls, number of incoming and outgoing SMS text messages, and the number of times screen is on
- Voice

#### Privacy Protection Adopted by Existing MMHS Studies

Not surprisingly, of the 32 surveyed studies, 11 (34%) did not mention any user privacy protection, as shown in Multimedia Appendix 1. This finding is in line with the findings of previous studies. For example, Nurgalieva et al [59] found that only a third of their reviewed mHealth papers considered privacy and security together. A recent survey study revealed that most (68%) of the reviewed MMHS were not sufficiently transparent regarding privacy protection information, whereas more than half had no privacy policy at all [60]. Furthermore, the study found that even in the case of mobile apps that had a privacy policy, researchers collected data without informing users about how the data would be used [60].

We categorized the user privacy protection mechanisms implemented in our surveyed studies into the following types: data anonymization; encryption (when transferring data from local mobile devices to remote data storage [eg, cloud storage]); access control; archiving only features extracted from the original data, instead of the original data; and allowing certain collected data to be wiped out remotely by users. Among them, data anonymization and encryption were the most common mechanisms used. A shared key is needed in the process of encryption and decryption, and, according to federal HIPAA and Health Information Technology for Economic and Clinical Health Act regulations, the key length must be 128 bits. The National Institute of Standards and Technology recommends using Suite-B, a set of algorithms that exchange decryption keys and digital signatures to authenticate data [9].

Despite the use of data anonymization and encryption having become common, there are still risks of data breach and disclosure, given that original data are stored physically. In comparison, archiving only selective features extracted from collected user data and allowing users to delete any collected data may help alleviate the risk of disclosure of, or unauthorized access to, personally sensitive data. For example, real-time audio processing can be used to extract relevant health inferences (ie, features) while discarding sensitive content. Of note, this option of privacy protection does not come without a cost—there is always a trade-off between user privacy and data utility: the fewer data points that MMHS collect, the higher the degree of user privacy protection but the more inferior the services they provide. For example, disabling collection of data about users’ physical activities or social interactions will help alleviate users’ privacy concerns, but it may also negatively affect the benefits of MMHS (eg, depression detection) because the systems may not infer users’ mental status accurately because of the removal of such data. By analogy, in e-commerce, consumers may sacrifice their privacy to some extent by allowing cookies to capture their behavior on an online retailer’s website to receive personalized services (eg, personalized product recommendations) [61]. Therefore, understanding user perceptions of different types of personal data with regard to privacy and developing and deploying effective privacy protection methods have the potential to break the value-privacy paradox, which will ultimately influence user adoptions and continuous use of MMHS.

#### The Research Model and Hypotheses

As summarized in the previous section, existing MMHS have collected various types of personal data. We investigate privacy concerns mainly from the 2 dimensions in this research: data type and data stage.

Researchers have found that the same individuals may have different levels of privacy concerns for different types of personal information [62]. For instance, online shoppers tend to be more likely to withhold information such as purchase history, social security number, hobbies, and favorite websites than name, gender, and education information [63]. Geographic location, mailing address, and information about friends and profession were common data types that a sample of 1000 French online users reported unwilling to disclose [64]. From a social media perspective, interaction with others through social networks usually leads to generating and sharing personal information actively [65]. Jin [66] suggests that although Twitter users often share personal information about their daily lives and entertainment choices, they would hardly reveal their own mental or physical health information. Thus, we propose the first hypothesis as follows:

Hypothesis 1. Data type has an effect on user privacy concerns with MMHS.

Data processing stages can be another critical dimension of privacy in MMHS. Data processing starts with data *collection*. Because of limited storage space as well as limited processing power of a mobile or wearable device (eg, smartphone), the data collected by MMHS are typically transferred to a remote server or to the cloud for processing and storage, which will finally lead to data sharing. Xu (2019) characterizes the combination of health informatics and cloud computing as Health Informatics as a Service [67]. Hindy et al (2020) emphasize the threats of personal information leakage at a data transmission level because mobile apps are increasingly dependent on wireless networks, which raises privacy concerns when transmitting data wirelessly [68]. Zeissig et al [69] and Kotz [33] suggest that privacy concerns vary with an app’s functionality and the entities that process data. Given that the data at different stages can be exposed to different levels of privacy risks and data transmission and data sharing are particularly vulnerable to intrusion with regard to data privacy, we propose the second hypothesis as follows:

Hypothesis 2. Data stage has an effect on user privacy concerns with MMHS.

Hypothesis 2.1. Privacy concerns for the (i) data transmission and (ii) data sharing stages are higher than those for the data collection stage.

Hypothesis 2.2. Privacy concerns for the (i) data transmission and (ii) data sharing stages are higher than those for the data storage stage.

Privacy awareness refers to the extent to which individuals are well informed about privacy practices and privacy breach incidents around themselves [70]. A number of studies have found that privacy awareness is positively associated with privacy concerns in the context of computer use [71], peer relationships on social media [72], older generation’s online privacy perception [69], personal cloud storage apps [73], news content ownership on social media [74], and so on.

Privacy victimization experience has been shown to influence privacy concerns in previous studies [70-72,75,76]. Privacy calculus theory [24] posits that individuals tend to weigh potential benefits and risks of data disclosure decisions. They will disclose personal information when the perceived benefits exceed the potential cost. If they have been previously victimized by privacy disclosure, they may perceive the cost of data disclosure to be higher than the benefit and be hesitant to take a risk. Therefore, previous experience of having been a victim of privacy intrusion could result in MMHS users assessing risks and foreseeing future consequences of privacy intrusion better. For example, Chen et al [77] suggest that online scam victims have higher perceived threat than nonvictims. Most victims of personal information breaches feel fearful, angry, and depressed after being victimized, leading to greater privacy concerns than before [78]. Bansal et al [75] suggest that privacy victimization experience would significantly increase when disclosing private information online. This positive relationship between privacy victimization experience and privacy concerns has also been demonstrated in e-commerce [76], internet use for general purposes [79], social network platforms [80,81], allowing permission requests for data acquisition by mobile apps [82], and Android app downloads [83]. Thus, we predict that people with a higher level of privacy awareness and privacy victimization experience would be more sensitive and concerned about privacy when using MMHS. Therefore, we propose the following 2 hypotheses:

Hypothesis 3. Privacy awareness is positively associated with privacy concerns about MMHS.

Hypothesis 4. Privacy victimization experience is positively associated with privacy concerns about MMHS.

An agreeable attitude toward privacy protection has been suggested as one of the major outcomes of privacy concerns [84,85]. For example, when an individual has a significant privacy concern, they would likely change their online account passwords more frequently than those with lower privacy concerns [77]. Deleting cookies, using ad blockers, and choosing a browser mode that keeps browsing history hidden are typical privacy protection methods used when browsing the web [86]. Similarly, users of social media [87,88] and e-commerce services [89] also seem to look for personal information protection after recognizing privacy risks.

Privacy literacy is another predictor of an agreeable attitude toward privacy protection. Self-control theory [77] posits that one’s ability to regulate emotions, behaviors, and desires is determined by one’s general intelligence and prior training. People who have high self-control derived from intelligence and sufficient training are likely to pursue a good way of solving a problem [90]. Accordingly, it is reasonable to predict that the level of a user’s privacy literacy, such as HIPAA knowledge level, may influence their privacy concerns about MMHS. Therefore, we hypothesize a positive relationship between HIPAA knowledge level and an agreeable attitude toward privacy protection as follows:

Hypothesis 5. Privacy concerns are positively associated with an agreeable attitude toward privacy protection in MMHS.

Hypothesis 6. HIPAA knowledge level is positively associated with an agreeable attitude toward privacy protection in MMHS.

Privacy protection methods can be viewed as solutions to coping with users’ privacy concerns. The Protection Motivation Theory explains how fear may change one’s attitude and behavior [91,92]. If an event incurs fear, one may try to reduce unstable emotional state and seek alternative ways in which one can find adaptive coping responses. In the context of MMHS, fear may arise from privacy concerns triggered by threats to personal information. Several studies have explored the relationship between the attitude toward privacy protection and intention to use mHealth systems [37,38]. Attitudes toward privacy protection involve a positive perception of usefulness and optimistic expectation of specific methods. It has been found that users’ perceived usefulness of health Internet of Things systems has a significant impact on their intention to use the systems [93]. For example, consumers tend to have a stronger willingness to use health recommendation systems when they feel that the latter are useful for fulfilling their health goals [94]. Employees’ optimistic attitudes toward their mobile devices have also been found to have a positive impact on users’ intention to use a mobile device in the workplace [95]. Therefore, we propose the following hypothesis:

Hypothesis 7. An agreeable attitude toward privacy protection is positively associated with the continuous usage intention of MMHS.

MMH literacy [96,97] plays an important role in the context of health care systems. Zhang and Yan [98] reported that eHealth literacy affected users’ continuous intention to use mHealth apps. Drawing on the Elaboration Likelihood Model [99], they suggested that eHealth literacy would foster satisfactory emotions for apps, which in turn motivated continuous intention to use them. Britt et al [100] demonstrated that a higher literacy level measured by the eHealth Literacy Scale led to a greater intention to use online health resources. In the same vein, Kim et al [101] found that mental health literacy would promote help-seeking behavior of college students. Therefore, we expect that patients with higher levels of MMH literacy may understand the potential benefits of MMHS better and accordingly are more likely to use them. Hence, we propose the following hypothesis:

Hypothesis 8. MMH literacy is positively associated with the continuous usage intention of MMHS.

**Figure 1:**
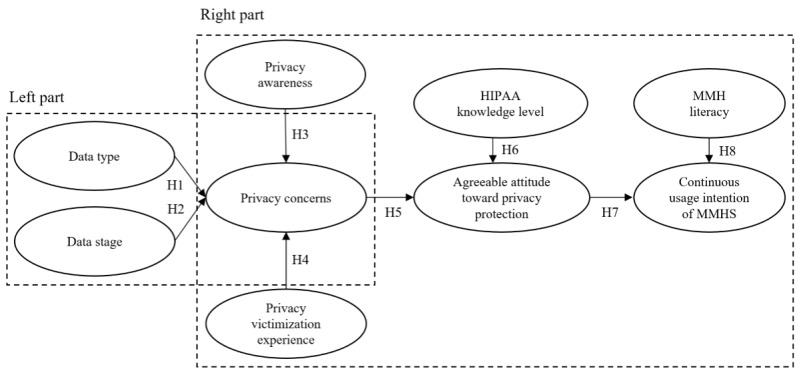
The research model (H: hypothesis; HIPAA: Health Insurance Portability and Accountability Act; MMH: mobile mental health; MMHS: mobile mental health systems).

Our research model is shown in Figure 1.

## Methods

To test the hypotheses, we conducted a web-based survey to collect data after receiving approval from the institutional review board of our institution.

### Survey Instruments and Procedure

Given that this study is targeted at a specific population, we deployed a prescreening questionnaire to determine participants’ eligibility for the study. The eligibility criteria were as follows: participants who (1) were aged ≥18 years, (2) had mental health issues in the past 12 months, and (3) had used any MMHS in the past 12 months. Only qualified participants could proceed with the formal survey.

The formal survey questionnaire (Multimedia Appendix 2) consisted of 3 parts: part 1 collected information about participants’ basic demographics, mHealth literacy, and knowledge about HIPAA; part 2 consisted of questions about participants’ use of MMHS and their prior experience with privacy protection methods; and part 3 asked questions about privacy concerns with regard to different data stages and data types. As discussed in the previous section, we considered the following 4 data stages: collection, transmission, storage, and sharing. In addition, we drew on the literature and identified the following 8 types of personal data: physiological signals, voice features, physical activities, facial expression, GPS location, social activities, device use, and self-reported data (Multimedia Appendix 1). Our design of the questionnaire for the agreeable attitude toward privacy protection, which was also based on the findings of our literature review (Multimedia Appendix 1), consisted of 11 items corresponding to the following privacy protection methods: (1) displaying privacy policy, (2) obtaining user consent, (3) disabling collection of personally identifiable data, (4) user control, (5) encryption, (6) secure data transmission, (7) restriction of data storage access, (8) location protection, (9) feature extraction from audio data, (10) feature extraction from text data, and (11) data retraction. All the survey questions were rated on a 7-point Likert scale, ranging from strongly disagree (score=1) to strongly agree (score=7), with a score of 4 being neutral. The details of the relevant questionnaire items are presented in Multimedia Appendix 3 [102-104] and Multimedia Appendix 4 [72,75,87,88,93,94,96,100,105,106].

To ensure data quality, we incorporated 3 attention-check questions into the survey, such as “Please skip this question and do not select anything.” We excluded from data analysis the data collected from the participants who failed to follow the instruction while responding to these questions.

### Participants

We recruited participants from multiple venues such as online mental health communities (eg, the depression community on Reddit [n=159], the anxiety community on Reddit [n=134], and the mental health group on Facebook [n=55]).

Among the 348 respondents who successfully passed the prescreening test questions, 134 (38.5%) failed the attention-check questions and another 44 (12.6%) completed the survey in an amount of time that was more than 3 SDs from the average time used by the participants of a pilot study. At the end, we obtained 48.9% (170/348) of valid responses. The demographic information of these respondents is presented in Table 1. Each participant was offered a US $5 Amazon gift card for completing the survey.

**Table 1:** Demographic statistics of the survey respondents (N=170).

Demographic characteristics		Participants, n (%)
**Age (years)**		
	18-25	47 (27.6)
	26-30	71 (41.7)
	31-35	42 (24.7)
	36-40	7 (4.1)
	41-45	3 (1.8)
**Gender**		
	Female	61 (35.9)
	Male	109 (64.1)
**Education**		
	High school graduate	3 (1.8)
	Some college	89 (52.4)
	College graduate	55 (32.4)
	Postgraduate degree	13 (7.6)
	Some postgraduate work	10 (5.9)
**Marital status**		
	Married	82 (48.2)
	Single	79 (46.5)
	Divorced	9 (5.3)

### Data Analysis

We tested the left part of the model using a 2-way repeated analysis of variance and deployed partial least squares (PLS) regression for the right part of the model (Figure 1) using SmartPLS software [107]. It is commonly recognized that correlations among independent variables might increase the variance and lower the power of regression analysis [108,109]. In view of the large number of data types considered in our research design, we first performed a principal component analysis through varimax rotation with Kaiser normalization [110] to identify the principal components based on the eigenvalues and corresponding eigenvectors of the covariance matrix. Next, based on the results, we selected 4 principal components that explained more than 82% of the variance of the original data types. Specifically, physiological signals, voice features, physical activities, and facial expressions were grouped together and labeled as *biometric factors*, whereas GPS location and social activities were grouped together and labeled as *social interactions*. The remaining original data types—*self-reported data* and *device use*—were left unchanged. These 4 data types were used in subsequent data analyses.

To support PLS regression analysis, we first examined the convergent validity and discriminant validity of the research constructs, which are critical building blocks of model evaluation. We tested the convergent validity with Cronbach α [111], composite reliability with rho_A [112], and discriminant validity with average variable extracted. Following the suggestion of Henseler et al [113], we further assessed the discriminant validity by applying the Heterotrait-Monotrait ratio of correlations. Correlations among the constructs are presented in Multimedia Appendix 5.

## Results

### Descriptive Statistics and Construct Validations

The test results of convergent and discriminant validity are reported in Table 2. They show that the internal consistency of all reflective constructs (ie, continuous usage intention, MMH literacy, privacy awareness, privacy victimization experience, and HIPAA knowledge level) was acceptable, with Cronbach α>.75. In addition, both their composite reliability and rho_A values exceed the cutoff threshold (0.70) [112]. The average variable extracted results show that all values were >0.60, the acceptable level [114]. The discriminant validity among the reflective constructs is further established based on the Heterotrait-Monotrait ratio of correlations (<0.90; Multimedia Appendix 6). The detailed factor loadings of the constructs and indicators are reported in Multimedia Appendix 7.

**Table 2:** Construct reliability and validity (reflective constructs only).

Constructs	Cronbach α	rho_A	Composite reliability	Average variable extracted
Continuous usage intention	.759	0.764	0.862	0.675
MMH^a^ literacy	.892	0.899	0.915	0.607
Privacy awareness	.829	0.834	0.886	0.660
Privacy victimization experience	.842	0.872	0.892	0.630
HIPAA^b^ knowledge level	1.000	1.000	1.000	1.000

^a^MMH: mobile mental health.

^b^HIPAA: Health Insurance Portability and Accountability Act.

The top 3 most common mental health issues of the participants based on their self-reports were depression (33), dysthymia (30), and anxiety (24). According to Wasil et al [115], there are approximately 325,000 mobile apps for health and wellness in the market (ie, Google Play and Apple App Store). Calm [116], Talkspace [117], PTSD (posttraumatic stress disorder) Coach [118], and Optimism [119] are the most commonly used MMHS among our survey respondents. Calm helps users practice meditation and sleep by providing mindfulness music and bedtime stories. It mainly collects data of users’ daily app use and time spent on meditating. Talkspace is designed to match a licensed mental health therapist with a user conveniently and affordably in comparison with in-person therapy. Talkspace allows users to submit text, image, and video data regarding their mental status when consulting therapists. PTSD Coach supports those who have PTSD. It gathers users’ self-assessment data of PTSD symptoms and daily app use data. Optimism is a mobile app used to track a user’s mood level on a daily basis as reported by users with mood disorder.

On the basis of the results of the principal component analysis, we identified 4 main personal data types with respect to privacy concerns, including the degree of privacy concerns arising from *biometric factors*, *social interactions*, *device use*, and *self-reported data*. The descriptive statistics of privacy concerns and other research constructs are reported in Tables 3 and 4, respectively. For all the variables in Tables 3 and 4, their median, maximum, and minimum values are 5, 7, and 1, respectively.

**Table 3:** Descriptive statistics of privacy concerns.

Research constructs and variables			Values, mean (SD)
**Data type**			
	Biometric factors	4.66 (1.89)	
	Social interaction	4.89 (1.71)	
	Device use	4.70 (1.76)	
	Self-reported data	4.92 (1.84)	
**Data stage**			
	Collect	4.61 (1.83)	
	Store	4.67 (1.83)	
	Transmit	4.80 (1.82)	
	Share	4.92 (1.82)	

**Table 4:** Descriptive statistics of other constructs.

Research constructs and variables	Values, mean (SD)
Privacy awareness	4.77 (1.75)
Privacy concerns (composite)	4.75 (1.83)
Privacy victimization experience	4.44 (1.96)
Agreeable attitude toward privacy protection (composite)	5.09 (1.57)
MMH^a^ literacy	4.84 (1.69)
HIPAA^b^ knowledge level	4.14 (1.92)
Continuous usage intention	5.04 (1.54)

^a^MMH: mobile mental health.

^b^HIPAA: Health Insurance Portability and Accountability Act.

### Effects of Data Type and Data Stage

We conducted a 2-way repeated analysis of variance by using privacy concerns as the dependent variable and data type and data stage as the independent variables. The results are reported in Table 5. The analyses yielded significant main effects of data type (*P*=.003) and data stage (*P*<.001), as well as their significant interaction effect (*P*=.008) on privacy concerns.

**Table 5:** Analysis of variance results for the effects of data type and data stage on privacy concerns.

Sources	*F* test (*df*)	Mean squared errors	*P* value
Data type	4.73 (3,507)	11.78	.003
Data stage	9.35 (3,507)	15.46	<.001
Data type×data stage	2.47 (9,1521)	2.25	.008

The results of post hoc multiple comparisons of the effects of data type and data stage are reported in Tables 6 and 7, respectively. The analysis results of data type show that social interaction data (*P*=.007) and self-reported data (*P*=.001) raise greater privacy concerns than biometrics data. In addition, social interaction data cause higher privacy concerns than device use data (*P*=.045), whereas device use data provoke privacy concerns more than self-reported data (*P*=.02).

**Table 6:** Results of comparison of privacy concerns across data types.

Data type (I) and data type (J)		Mean difference (I–J)	*P* value	SE
**Biometrics factors**				
	Social interaction	–0.232	.007	0.084
	Device use	–0.044	.53	0.070
	Self-reported data	–0.262	.001	0.079
**Social interaction**				
	Device use	0.188	.045	0.093
	Self-reported data	–0.030	.74	0.092
**Device use**				
	Self-reported data	–0.218	.02	0.093

**Table 7:** Results of comparison of privacy concerns across data stages.

Stage (I) and stage (J)			Mean difference (I–J)		*P* value		SE
**Collect**							
	Store	–0.056		.36		0.060	
	Transmit	–0.197		.01		0.075	
	Share	–0.336		<.001		0.091	
**Store**							
	Transmit	–0.142		.01		0.057	
	Share	–0.281		<.001		0.068	
**Transmit**							
	Share	–0.139		.02		0.061	

The analysis results of data stage show that the data transmission stage raises greater privacy concerns than both data collection (*P*=.01) and data storage stages (*P*=.01), whereas the data sharing stage also raises higher privacy concerns than the data collection (*P*<.001), data storage (*P*<.001), and data transmission stages (*P*=.02). However, no difference was detected between the data collection and data storage stages (*P*=.36). Thus, hypotheses 1, 2.1 (i), 2.1 (ii), 2.2 (i), and 2.2 (ii) are supported.

### Effects on Continuous Usage Intention

**Figure 2:**
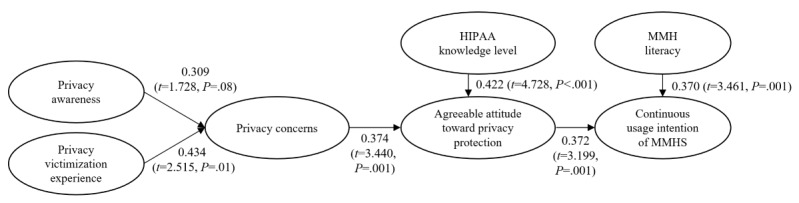
Results of the research model. HIPAA: Health Insurance Portability and Accountability Act; MMH: mobile mental health; MMHS: mobile mental health systems.

The results of PLS regression analysis are reported in Table 8 and Figure 2. The results show that privacy victimization experience (*P*=.01) has a significant effect, whereas privacy awareness has a marginally significant effect (*P*=.08) on privacy concerns. Therefore, hypothesis 3 is marginally supported, whereas hypothesis 4 is supported. In addition, both privacy concerns (*P*=.001) and HIPAA knowledge level (*P*<.001) have a positive effect on agreeable attitude toward privacy protection. Therefore, both hypotheses 5 and 6 are supported. Furthermore, both agreeable attitude toward privacy protection (*P*=.001) and MMH literacy (*P=*.001) have a positive effect on the continuous usage intention of MMHS. Therefore, hypotheses 7 and 8 are also supported.

**Table 8:** Results of partial least squares regression analysis.

Hypotheses	Estimate (SD)	t-statistic (*df*)	*P* value
Data type→Privacy concerns	—^a^	—^a^	—^a^
Data transmission and data sharing stages→Privacy concerns	—^b^	—^b^	—^b^
Privacy awareness→Privacy concerns	0.309 (0.179)	1.728 (499)	.08
Privacy victimization experience→Privacy concerns	0.434 (0.172)	2.515 (499)	.01
Privacy concerns→Agreeable attitude toward privacy protection	0.374 (0.109)	3.440 (499)	.001
HIPAA^c^ knowledge level→Agreeable attitude toward privacy protection	0.422 (0.089)	4.728 (499)	<.001
Agreeable attitude toward privacy protection→Continuous usage intention of MMHS^d^	0.372 (0.116)	3.199 (499)	.001
MMH^e^ literacy→Continuous usage intention of MMHS	0.370 (0.107)	3.461 (499)	.001

^a^See Table 6.

^b^See Table 7.

^c^HIPAA: Health Insurance Portability and Accountability Act.

^d^MMHS: mobile mental health systems.

^e^MMH: mobile mental health.

## Discussion

### Overview

MMHS have been increasingly used to monitor users’ emotional status, improve mental illness management, and retain psychological well-being [120]. However, users’ privacy concerns with regard to the use of MMHS can be a critical barrier to their adoption of, and benefiting from, these systems [121]. This study proposes and tests a novel research model for explaining user privacy concerns about MMHS from the data and user experience perspectives and examines the direct or indirect effects of privacy concerns on user perceptions of different privacy protection methods and intention to continue using MMHS.

### Principal Findings

First, we discovered a significant main effect of data type on privacy concerns. Respondents expressed stronger privacy concerns about social interaction data (eg, outgoing or incoming phone calls and SMS text messages) and self-reported data (eg, current medication) than physiological data and device use data. The strong concern about social interaction data is somewhat surprising because one may assume intuitively that physiological signals (ie, skin temperature and heart rate) and physical activities (ie, walking and sleeping) should be more privacy sensitive. A possible explanation is that social isolation is one of the most typical characteristics of individuals with mental health issues [122,123]. As a result, this subpopulation may perceive social interaction data as more private than physiological and physical activity data.

Second, this study reveals a significant effect of data stage on privacy concerns. Specifically, data transmission and data sharing cause higher privacy concerns than data collection and data storage, which supports our hypotheses.

Third, the results confirm our hypothesis that privacy victimization experience has a positive effect on privacy concerns. Although privacy awareness is positively associated with privacy concerns, this effect was only marginally significant at a 0.1 significance level. A possible explanation lies in what constitutes privacy awareness. Correia and Compeau [124] argue that privacy awareness consists of 3 elements: the literacy of the elements related to privacy, the recognition that the elements exist in a current system, and the forecast of their impacts on the future. Thus, these aspects may guide future efforts in improving the effectiveness of privacy awareness training.

Fourth, the findings of this study show that increasing privacy concerns escalate agreeable attitude toward privacy protection.

Fifth, our findings show that privacy knowledge about HIPAA contributes to an agreeable attitude toward privacy protection of MMHS. In addition, MMH literacy facilitates continuous usage intention of using MMHS. The findings suggest the importance of increasing privacy knowledge and mHealth literacy of users with mental health issues for improving the use of MMHS.

### Research Contributions

Despite increasing efforts being made with regard to leveraging mobile and sensing technologies for improving public mental health, there has been a lack of research on the understanding of users’ privacy concerns and their impacts on the use of MMHS. This study makes contributions to the multidisciplinary literature. First, to the best of our knowledge, this study is the first research effort that systematically investigates user privacy concerns in the context of MMHS. Second, this study not only extends the Antecedents→Privacy Concerns→Outcomes model to MMHS, but also introduces new constructs, including HIPAA knowledge level and MMH literacy. Third, unlike prior studies that treated privacy data as monotonic [86,125,126], this research for the first time innovatively probes different data types and stages while investigating privacy concerns. The differences in the effects on privacy concerns of different data types and data stages have significant implications for future privacy research. Fourth, this study introduces MMH literacy as an antecedent to the continuous usage intention of MMHS. eHealth literacy has been used to assess healthy behavior on the internet [127-129], but it has rarely been used to explain the continuous intention to use innovative technology. Last but not least, this study goes beyond privacy concerns by understanding their effects on privacy protection. Our research findings reveal that an agreeable attitude toward privacy protection mediates the relationship between privacy concerns and users’ continuous MMHS usage intention.

### Practical Implications

This study offers a number of practical implications for different stakeholders of MMHS. For designers and developers of user-centric privacy-protecting MMHS, different effects of various personal data on privacy concerns suggest that different types of personal data should not be treated equally from a privacy protection perspective; designers and developers should care not only about the types of user data being collected, but also about how the data will be processed. In particular, they should pay more attention to effective privacy protection methods deployed for data sharing and data transmission than those deployed for data collection and data storage. As users differ in terms of their sensitivity to privacy and different personal data, the deployed privacy protection methods should be user-centered and personalized; the effect of privacy concerns on continuous MMHS usage intention can be mediated by privacy protection. Thus, implementing privacy protection measures and developing ways to improve the MMH literacy of patients can be effective strategies for increasing the trust of patients with mental health issues in MMHS and their adoption and continuous use of MMHS.

From an MMHS user perspective, users should increase their awareness of different types of data collected by MMHS; improve their knowledge regarding privacy and MMH literacy; and be educated about different privacy protection methods, which can help them choose MMHS and understand how these methods can possibly address their privacy concerns.

The following is a set of general guidelines for the design of user-centered, privacy-preserving MMHS based on the findings of this research:

- Only collect user data that are relevant to MMH
- Deidentify any data that may reveal the identity of individual users
- Encrypt data, particularly during data transmission and data sharing
- Provide user-controlled data collection, enabling users to remove certain collected data of their choice
- Provide user-controlled data access: data access and sharing should be limited to specific, user-approved parties
- Provide continuous mobile user authentication to ensure that the data are collected from the right person
- Include audits and risk assessment in privacy protocols
- Set up a policy that encrypts self-reported data and social interaction data
- Collect information about users’ prior experiences of privacy victimization and recommend targeted privacy protection methods
- Improve public education about the goals, methods, and procedures of data management and privacy protection, which is essential
- Allow users to adjust privacy levels and retract collected data. This will be one of the balanced solutions in practice
- Design MMHS with an emphasis on personalized privacy protection. Personalization is one of the key features of recent MMHS to provide customized treatment to individuals

### Limitations of the Study and Future Research

This study includes several limitations that offer future research opportunities. We used a web-based survey for data collection in this study, which is subject to the limitations of self-reported data. Future studies may collect actual patient use data by either collaborating with MMHS providers or using self-developed mobile apps. We also acknowledge that our recruitment strategy may pose a potential risk for selection bias—though our university-wide solicitation for participation was circulated through the university’s email listserv, students could be more technology savvy and therefore more willing to participate than faculty and staff members. In addition, our recruitment flyer, which was circulated through online mental health communities, may have caused hesitation among individuals who have privacy concerns about using technology for mental health to participate in this survey. In addition to data type and data stage, other factors of MMHS, such as system functions, can be potential antecedents of privacy concerns. For instance, MMHS that focus on improving mindfulness and sleep quality, such as Calm and Headspace [130,131], are likely to yield different levels of privacy concerns compared with MMHS that focus on serious clinical mental illness, such as PTSD Coach and NOCD (an MMHS for the treatment of obsessive-compulsive disorder).

## Appendix

Multimedia Appendix 1 A summary of collected patient data and privacy protection methods in existing mobile mental health studies (N=32).

Multimedia Appendix 2 Survey questionnaire.

Multimedia Appendix 3 Construct measurement - privacy concerns.

Multimedia Appendix 4 Construct measurements - other concerns.

Multimedia Appendix 5 Correlations among the constructs.

Multimedia Appendix 6 Discriminant validity: Heterotrait-Monotrait ratio of correlations.

Multimedia Appendix 7 Factor loadings.

## Abbreviations

HIPAA: Health Insurance Portability and Accountability Act
mHealth: mobile health
MMH: mobile mental health
MMHS: mobile mental health systems
PHI: protected health information
PLS: partial least squares
