# Supplementary material for: Breaking the Data Value-Privacy Paradox in Mobile Mental Health Systems Through User-Centered Privacy Protection: A Web-Based Survey Study
Source: JMIR Ment Health. 2021 Dec 24;8(12):e31633. doi: 10.2196/31633 (PMC8742208; doi:10.2196/31633)
Supplement: Multimedia Appendix 1 [file mental_v8i12e31633_app1.docx]

**Multimedia Appendix 1.** A summary of collected patient data and privacy protection methods in existing mobile mental health studies (N=32).

| Mental health issues | Data collected | Privacy protection | Study |
| --- | --- | --- | --- |
| **Parkinson disease** | | | |
|  | Tremor at rest, gait, finger tapping, activity time, sleep, and so on | Data encryption for transmission; access control | Tsiouris et al [38] |
|  | Keystroke features on touchscreens | None | Arroyo-Gallego et al [39] |
| Obstructive pulmonary disease | Vital signs (biomarkers): oxygen saturation, heart rate, temperature, peak expiratory flow rate, and walking distance | None | Bitsaki et al [40] |
| **Depression** | | | |
|  | GPS features, including location variance, entropy, and circadian movement | None | Saeb et al [41] |
|  | Ecological momentary assessment and GPS data | Data anonymization and encryption | Saeb et al [42] |
|  | Question-answer pairs during patient-chatbot communication (chat-based intervention) | Users enter their own dialog | Delahunty et al [43] |
|  | Activity, sleep, and heart rate by Fitbit; categorical GPS data | Data anonymization | Bidja [4] |
| **Bipolar disorder** | | | |
|  | Number of apps used by the user, number of times screen is on, the time that the user spends on their smartphone, and so on | None | Alvarez-Lozano et al [44] |
|  | Phone call behavior (eg, number and length of phone calls) and sound features (eg, numbers of speaker turns and short turns or utterances) | Data anonymity | Grünerbl et al [45] |
|  | Location, distance traveled, conversation frequency, bedtime, waking time, nonstationary duration, and so on | No recording of audio content; only extracted and stored audio features (eg, spectral content and regularity and loudness) | Abdullah et al [19] |
|  | Duration of a keypress, time since last keypress, distance from last key along 2 axes, and accelerometer movement | Individual character data, with the exceptions of the backspace key and space bar | Cao et al [5] |
|  | Numbers and durations of incoming and outgoing phone calls per day; numbers of incoming and outgoing text messages | None | Faurholt‐Jepsen et al [46] |
| **Schizophrenia** | | | |
|  | Distance traveled, sleep and conversation durations, physical activities, audio amplitude, accelerometer readings, light sensor readings, location coordinates, app use, and so on | Data uploaded to a secured server using encrypted SSL^a^ connections | Wang et al [47] |
|  | Speech features (eg, energy and relative spectral entropy), physical activities, and location | Not the original speech content but speech features only | Ben-Zeev et al [48] |
|  | Physical activities, sleep (eg, duration and bedtime), sociability (ie, the number of independent conservations a participant is around and duration), audio amplitude, accelerometer readings, light sensor readings, location coordinates, app use, and call logs | No collection of phone numbers, content of text messages, or any conversation content | Wang et al [6] |
|  | Sleep quality (acceleration, heart rate, ambient lighting, battery level and charging events, screen on and off) and self-rated symptoms (diaries) | Data encryption; no personally identifiable digital information | Meyer et al [16] |
|  | Time using their mobile devices, texting, gaming, internet browsing, using the camera, and so on, and qualitative responses to surveys (eg, Brief Symptom Inventory) | Data encryption and access control; data security and compliance with national guidelines and regulations | Kidd et al [15] |
| **Mental state sensing and well-being** | | | |
|  | Accelerometer data, skin temperature and conductance, number of calls, SMS text messages, location, internet use, and *screen on* timing | None | Sano et al [49] |
|  | Heart rate, respiration rate, ECG^b^ signal, blood pressure, and oxygen saturation | None | Naddeo et al [50] |
|  | ECG, heart rate variability, respiration activity, and physical activities | None | Lanata et al [51] |
|  | Skin temperature, galvanic skin resistance, and heart rate variability | Encryption of data for transmission | Fraiwan [17] |
|  | Bluetooth use, location, and phone battery status | Cryptographically hashed | Boonstra [52] |
|  | Audio features (entropy, energy, 5 formants, number of formants more than 0, brightness, nonzero formants’ average, formants’ mean, sum, and speaking time), activity (accelerometer and gyroscope data), and social features (questionnaires) | Feature extraction to avoid leakage of private information before transmission | Yang et al [18] |
|  | Physiological signals (eg, heart rate, heart rate variability, and skin conductance) | None | Birenboim et al [12] |
| Mood detection | Keystroke features (eg, average interkey delay, backspace ratio, average session length, and session count) | Data encryption for uploading | Zulueta et al [53] |
| **Dementia** | | | |
|  | Real-time pictures along a user’s walking path and GPS data | Data encryption and decryption; whenever the phone is activated, the system will activate the front camera to take a picture of the current user and compare it with the owner’s prestored photo to authenticate the user | Ko et al [54] |
|  | Measurement of gait (eg, stride length, stride time, cadence, velocity, step length asymmetry, and step time asymmetry) and balance through ambient technology installed in a specialized inpatient unit | Using RFID^c^ to identify individual participants | Dolatabadi et al [55] |
| **Stress** | | | |
|  | Skin conductance and temperature, location, number of naps, timing of calls and SMS text messages, mobility patterns, and phone screen–on time | No recording of the content of emails, calls, or SMS text messages | Sano et al [56] |
|  | Heart rate, skin temperature, and galvanic skin response (ie, measuring the perspiration or electro dermal activity of the skin) | None | Salafi and Kah [13] |
|  | Mobile phone activities (eg, phone calls and SMS text messages), proximity of nearby phones or devices, and so on | None | Bogomolov et al [14] |
|  | Phone use data of 6 app categories: social (eg, Skype and social networks), entertainment (games and music), infotainment (news and books), business (calendar and editing), well-being (weight watching and exercise monitoring), and any other interaction with a phone | Accumulative data about app use; contents of the webpages or keystrokes were not logged | Vildjiounaite et al [57] |
| Autism | Facial expression and corresponding emotions of patients captured by Google Glass | Providing families an option to delete videos during an intervention period | Voss et al [58] |

^a^SSL: Secure Sockets Layer.

^b^ECG: electrocardiogram.

^c^RFID: radio frequency identification.
