# Supplementary material for: Breaking the Data Value-Privacy Paradox in Mobile Mental Health Systems Through User-Centered Privacy Protection: A Web-Based Survey Study
Source: JMIR Ment Health. 2021 Dec 24;8(12):e31633. doi: 10.2196/31633 (PMC8742208; doi:10.2196/31633)
Supplement: Multimedia Appendix 2 [file mental_v8i12e31633_app2.docx]

**Multimedia Appendix 2. Survey questionnaire**

**Section A. Demographics and knowledge level**

1. What is your gender?

- Female
- Male

2. What is your age? ( )

3. What is your highest degree of school completed or in progress?

- Some high school
- High school graduate
- Some college
- College graduate
- Some post graduate work
- Post graduate degree

4. What is your marital status?

- Single
- Married
- Widowed
- Divorced
- Separated

5. Please indicate to what extent you agree with each of the following statements.

*Scale (Strongly disagree – Disagree - Somewhat disagree - Neutral – Somewhat agree - Agree - Strongly agree)

| I know what health resources are available on a mobile mental health app. |
| --- |
| I know where to find helpful health resources on a mobile mental health app. |
| I know how to use the information I find on a mobile mental health app to help me. |
| I know how to find helpful resources on a mobile mental health app. |
| I have the skills needed to evaluate the resources I find on a mobile mental health app. |
| I know how to use a mobile mental health app to answer my questions about health. |
| I feel confident in using information from a mobile mental health app to make decisions. |

6. Please indicate to what extent you are knowledgeable about HIPAA (Health Insurance Portability and Accountability Act) Privacy Rule.

- Not knowledgeable at all
- Poorly knowledgeable
- Slightly knowledgeable
- Moderately knowledgeable
- Knowledgeable
- Very knowledgeable
- Extremely knowledgeable

**Section B. Experience with the mobile mental health app(s)**

1. Specify the name of the app that you used most frequently in the past 12 months.

2. How long have you been using the app?

3. How often do (did) you use the app?

- Multiple times a day
- Once a day
- Once every few days
- Once a week
- A few times a month
- A few times a year

**Section C. Privacy Concerns**

*Scale (Strongly disagree – Disagree - Somewhat disagree - Neutral – Somewhat agree - Agree - Strongly agree)

1. A mobile mental health app may COLLECT various detailed data from you. Please rate your privacy concern with having each of the following types of data collected by an app.

*I am concerned about the app collecting detailed data about my __________.*

| physiological signals (e.g., skin temperature, heart rate) |
| --- |
| device usage and interaction (e.g., time of accessing a mobile device) |
| GPS Location information (e.g., latitude, longitude) |
| social activities (e.g., duration of a phone call, timestamp of text messages) |
| voice features (e.g., pitch, sharpness) |
| physical activities (e.g., walk time, walk speed) |
| facial expression (e.g., mouth open, eyebrow raise) |
| self-reported data (e.g., weight, type of medication taking) |

2. A mobile mental health app may STORE your detailed data in the cloud. Please rate your privacy concern with storing each of the following types of data in the cloud.

*I am concerned about the app collecting detailed data about my __________.*

| physiological signals (e.g., skin temperature, heart rate) |
| --- |
| device usage and interaction (e.g., time of accessing a mobile device) |
| GPS Location information (e.g., latitude, longitude) |
| social activities (e.g., duration of a phone call, timestamp of text messages) |
| voice features (e.g., pitch, sharpness) |
| physical activities (e.g., walk time, walk speed) |
| facial expression (e.g., mouth open, eyebrow raise) |
| self-reported data (e.g., weight, type of medication taking) |

3. A mobile mental health app may automatically TRANSMIT your detailed data to the service provider. Please rate your privacy concern with transmitting each of the following types of data.

*I am concerned about the app collecting detailed data about my __________.*

| physiological signals (e.g., skin temperature, heart rate) |
| --- |
| device usage and interaction (e.g., time of accessing a mobile device) |
| GPS Location information (e.g., latitude, longitude) |
| social activities (e.g., duration of a phone call, timestamp of text messages) |
| voice features (e.g., pitch, sharpness) |
| physical activities (e.g., walk time, walk speed) |
| facial expression (e.g., mouth open, eyebrow raise) |
| self-reported data (e.g., weight, type of medication taking) |

4. A mobile mental health app may SHARE the detailed data collected from you with the third party. Please rate your privacy concern about sharing each of the following types of data.

*I am concerned about the app collecting detailed data about my __________.*

| physiological signals (e.g., skin temperature, heart rate) |
| --- |
| device usage and interaction (e.g., time of accessing a mobile device) |
| GPS Location information (e.g., latitude, longitude) |
| social activities (e.g., duration of a phone call, timestamp of text messages) |
| voice features (e.g., pitch, sharpness) |
| physical activities (e.g., walk time, walk speed) |
| facial expression (e.g., mouth open, eyebrow raise) |
| self-reported data (e.g., weight, type of medication taking) |

5. The following statements are about your attitude toward each privacy protection mechanism when using a mobile mental health app. Please indicate to what extent you agree or disagree with each statement.

| Statements |
| --- |
| If a mobile mental health app can display a policy on how to protect my personal data in the app, which might be shown to me right as I am downloading or signing up for the app, it would address my privacy concerns. |
| If a mobile mental health app needs my consent for how my personal data will be collected and protected in a mobile mental health app, which might have occurred right after I review the privacy policy, it would address my privacy concerns. |
| If a mobile mental health app is designed to prevent it from collecting identifiable data, it would help protect my privacy. |
| If a mobile mental health app enables me to change its data collection method in my preferred way, it would help protect my privacy. |
| If the app notifies me that the collected data from me will be encrypted safely, it would help protect my privacy. |
| If my collected data would be transmitted to the remote database securely, it would help protect my privacy. |
| If a mobile mental health app restricts others’ access to the data collected from me, it would help protect my privacy. |
| If a mobile mental health app protects my real-world location information, it would help protect my privacy. |
| Extracting and storing features of audio, not the original audio data collected from me, would help protect my privacy. |
| Extracting and storing features (e.g., emotions expressed in the text) of text that I generate (e.g., the text messages I sent or social media content I post), not the original content of textual content I generate, would help protect my privacy. |
| If a mobile mental health app allows me to remove any part of data already collected from me, it would help to protect my privacy. |

6. The following statements are about your intention to continue using a mobile mental health app. Please indicate to what extent you agree with each statement.

| Statements |
| --- |
| I intend to continuously use the app for my mental issue. |
| I predict that I will continuously use the app to improve my mental issue. |
| I plan to use the app for my mental issue continuously as part of my daily routine. |

7. The following statements are about your privacy victimization experience in general. Please indicate to what extent you agree with each statement.

| Statement |
| --- |
| I have frequently been the victim of an improper invasion of my information privacy by someone I know. |
| Only rarely is my information privacy invaded by someone I know. |
| I often feel that my information privacy has been being violated by someone I know. |
| My information privacy is invaded all the time by other people I know. |
| People I know often misuse my private information. |

8. The following statements are about your privacy awareness in general. Please indicate to what extent you agree with each statement.

| Statement |
| --- |
| Almost every day, I hear something about the invasion of someone’s information privacy by people they know. |
| I frequently hear about the invasion of someone’s information privacy by people they know. |
| There is often news about how someone misuses information regarding a person she or he knows. |
| People often share information they should not about someone they know. |
