# Supplementary material for: Breaking the Data Value-Privacy Paradox in Mobile Mental Health Systems Through User-Centered Privacy Protection: A Web-Based Survey Study
Source: JMIR Ment Health. 2021 Dec 24;8(12):e31633. doi: 10.2196/31633 (PMC8742208; doi:10.2196/31633)
Supplement: Multimedia Appendix 3 [file mental_v8i12e31633_app3.docx]

## Multimedia Appendix 3. Construct measurement - privacy concerns

| Latent variable | Indicator | PCA variables | Data types | Items |
| --- | --- | --- | --- | --- |
| Privacy Concerns (PC) [102-104] | PC1 | Biometric Factor | physiological signals | I am concerned about the app collecting detailed data about my physiological signals (e.g., skin temperature, heart rate). |
|  |  |  | voice features | I am concerned about the app collecting detailed data about my voice features (e.g., pitch, sharpness). |
|  |  |  | physical activities | I am concerned about the app collecting detailed data about my physical activities (e.g., walk time, walk speed). |
|  |  |  | facial expression data | I am concerned about the app collecting detailed data about my facial expression (e.g., mouth open, eyebrow raise). |
|  | PC2 | Social Interaction | GPS location | I am concerned about the app collecting detailed data about my GPS Location information (e.g., latitude, longitude). |
|  |  |  | social activities data | I am concerned about the app collecting detailed data about my social activities (e.g., duration of a phone call, timestamp of text messages). |
|  | PC3 | Device Usage | device usage | I am concerned about the app collecting detailed data about my device usage and interaction (e.g., time of accessing a mobile device). |
|  | PC4 | Self-reported Data | self-reported data | I am concerned about the app collecting detailed data about my self-reported data (e.g., weight, type of medication taking). |

PCA: Principal Component Analysis
