# Supplementary material for: Breaking the Data Value-Privacy Paradox in Mobile Mental Health Systems Through User-Centered Privacy Protection: A Web-Based Survey Study
Source: JMIR Ment Health. 2021 Dec 24;8(12):e31633. doi: 10.2196/31633 (PMC8742208; doi:10.2196/31633)
Supplement: Multimedia Appendix 4 [file mental_v8i12e31633_app4.docx]

## Multimedia Appendix 4. Construct measurement – others

| Latent variables | Indicators | Items |
| --- | --- | --- |
| Privacy Awareness (PA) [72, 105] | PA1 | Almost every day, I hear something about the invasion of someone’s information privacy by people they know. |
|  | PA2 | I frequently hear about the invasion of someone’s information privacy by people they know. |
|  | PA3 | There is often news about how someone misuses information regarding a person she or he knows. |
|  | PA4 | People often share information they should not worry about someone they know. |
| Privacy Victimization Experience (PVE) [72, 75] | PVE1 | I have frequently been the victim of an improper invasion of my information privacy by someone I know. |
|  | PVE2 | Only rarely is my information privacy invaded by someone I know. |
|  | PVE3 | I often feel that my information privacy has been being violated by someone I know. |
|  | PVE4 | My information privacy is invaded all the time by other people I know. |
|  | PVE5 | People I know often misuse my private information. |
| HIPAA Knowledge Level (HKL) [106] | HKL | Please indicate to what extent you are knowledgeable about HIPAA (Health Insurance Portability and Accountability Act) Privacy Rule. |
| Mobile Mental Health Literacy (MMHL) [96, 100] | MMHL1 | I know what health resources are available on a mobile mental health app. |
|  | MMHL2 | I know where to find helpful health resources on a mobile mental health app. |
|  | MMHL3 | I know how to use the information I find on a mobile mental health app to help me. |
|  | MMHL4 | I know how to find helpful resources on a mobile mental health app. |
|  | MMHL5 | I have the skills needed to evaluate the resources I find on a mobile mental health app. |
|  | MMHL6 | I know how to use a mobile mental health app to answer my questions about health. |
|  | MMHL7 | I feel confident in using information from a mobile mental health app to make decisions. |
| Agreeable Attitude Toward Privacy Protection (AAPP) [87, 88] | AAPP1 (Displaying policy) | If a mobile mental health app can display a policy on how to protect my personal data in the app, which might be shown to me right as I am downloading or signing up for the app, it would address my privacy concerns. |
|  | AAPP2 (Obtaining consent) | If a mobile mental health app needs my consent for how my personal data will be collected and protected in a mobile mental health app, which may occur right after I review the privacy policy, it would address my privacy concerns. |
|  | AAPP3 (Prevent collecting identifiable data) | If a mobile mental health app is designed to prevent it from collecting identifiable data, it would help protect my privacy. |
|  | AAPP4 (User control) | If a mobile mental health app enables me to change its data collection method in my preferred way, it would help protect my privacy. |
|  | AAPP5 (Encryption) | If the app notifies me that the collected data from me will be encrypted safely, it would help protect my privacy. |
|  | AAPP6 (Secure data transmission) | If my collected data would be transmitted to the remote database securely, it would help protect my privacy. |
|  | AAPPM7 (Restriction of data storage access) | If a mobile mental health app restricts others’ access to the data collected from me, it would help protect my privacy. |
|  | AAPPM8 (Location protection) | If a mobile mental health app protects my real-world location information, it would help protect my privacy. |
|  | AAPPM9 (Audio data feature extraction) | Extracting and storing features of audio, not the original audio data collected from me would help protect my privacy. |
|  | AAPPM10 (Text data feature extraction) | Extracting and storing features (e.g., emotions expressed in the text) of text that I generate (e.g., the text messages I sent or social media content I post), not the original content of textual content I generate, would help protect my privacy. |
|  | AAPPM11 (Data retraction) | If a mobile mental health app allows me to remove any part of data already collected from me, it would help to protect my privacy. |
| Continuous Usage Intention (CUI) [93, 94] | CUI1 | I intend to continuously use the app for my mental issue. |
|  | CUI2 | I predict that I will continuously use the app to improve my mental issue. |
|  | CUI3 | I plan to use the app for my mental issue continuously as part of my daily routine. |
