# Supplementary material for: Breaking the Data Value-Privacy Paradox in Mobile Mental Health Systems Through User-Centered Privacy Protection: A Web-Based Survey Study
Source: JMIR Ment Health. 2021 Dec 24;8(12):e31633. doi: 10.2196/31633 (PMC8742208; doi:10.2196/31633)
Supplement: Multimedia Appendix 5 [file mental_v8i12e31633_app5.docx]

## Multimedia Appendix 5. Correlations among the constructs

|  | AAPPM | HKL | CUI | MMHL | PA | PC | PVE |
| --- | --- | --- | --- | --- | --- | --- | --- |
| AAPPM | 1.000 | 0.558 | 0.628 | 0.691 | 0.569 | 0.528 | 0.452 |
| HKL | 0.558 | 1.000 | 0.556 | 0.504 | 0.458 | 0.364 | 0.371 |
| CUI | 0.628 | 0.556 | 1.000 | 0.627 | 0.536 | 0.446 | 0.446 |
| MMHL | 0.691 | 0.504 | 0.627 | 1.000 | 0.504 | 0.545 | 0.494 |
| PA | 0.569 | 0.458 | 0.536 | 0.504 | 1.000 | 0.640 | 0.762 |
| PC | 0.528 | 0.364 | 0.446 | 0.545 | 0.640 | 1.000 | 0.669 |
| PVE | 0.452 | 0.371 | 0.446 | 0.494 | 0.762 | 0.669 | 1.000 |
