# Supplementary material for: Breaking the Data Value-Privacy Paradox in Mobile Mental Health Systems Through User-Centered Privacy Protection: A Web-Based Survey Study
Source: JMIR Ment Health. 2021 Dec 24;8(12):e31633. doi: 10.2196/31633 (PMC8742208; doi:10.2196/31633)
Supplement: Multimedia Appendix 6 [file mental_v8i12e31633_app6.docx]

## Multimedia Appendix 6. Discriminant validity: Heterotrait-Monotrait ratio of correlations

|  | HKL | CUI | MMHL |
| --- | --- | --- | --- |
| HKL | - | - | - |
| CUI | 0.640 | - | - |
| MMHL | 0.528 | 0.752 | - |
| PA | 0.495 | 0.674 | 0.581 |
| PVE | 0.420 | 0.582 | 0.574 |
