# Supplementary material for: Breaking the Data Value-Privacy Paradox in Mobile Mental Health Systems Through User-Centered Privacy Protection: A Web-Based Survey Study
Source: JMIR Ment Health. 2021 Dec 24;8(12):e31633. doi: 10.2196/31633 (PMC8742208; doi:10.2196/31633)
Supplement: Multimedia Appendix 7 [file mental_v8i12e31633_app7.docx]

## Multimedia Appendix 7. Factor loadings

| Latent variables | Indicators | AAPP | HKL | CUI | MMHL | PA | PC | PVE |
| --- | --- | --- | --- | --- | --- | --- | --- | --- |
| Mobile Mental Health Literacy | MMHL1 |  |  |  | 0.808 |  |  |  |
|  | MMHL2 |  |  |  | 0.801 |  |  |  |
|  | MMHL3 |  |  |  | 0.781 |  |  |  |
|  | MMHL4 |  |  |  | 0.774 |  |  |  |
|  | MMHL5 |  |  |  | 0.73 |  |  |  |
|  | MMHL6 |  |  |  | 0.741 |  |  |  |
|  | MMHL7 |  |  |  | 0.816 |  |  |  |
| Continuous Usage Intention | CUI1 |  |  | 0.768 |  |  |  |  |
|  | CUI2 |  |  | 0.852 |  |  |  |  |
|  | CUI3 |  |  | 0.843 |  |  |  |  |
| Privacy Victimization Experience | PVE1 |  |  |  |  |  |  | 0.865 |
|  | PVE2 |  |  |  |  |  |  | 0.506 |
|  | PVE3 |  |  |  |  |  |  | 0.787 |
|  | PVE4 |  |  |  |  |  |  | 0.874 |
|  | PVE5 |  |  |  |  |  |  | 0.872 |
| Privacy Awareness | PA1 |  |  |  |  | 0.811 |  |  |
|  | PA2 |  |  |  |  | 0.851 |  |  |
|  | PA3 |  |  |  |  | 0.808 |  |  |
|  | PA4 |  |  |  |  | 0.78 |  |  |
| Agreeable Attitude toward Privacy Protection | AAPP1 | 0.818 |  |  |  |  |  |  |
|  | AAPP2 | 0.748 |  |  |  |  |  |  |
|  | AAPP3 | 0.455 |  |  |  |  |  |  |
|  | AAPP4 | 0.34 |  |  |  |  |  |  |
|  | AAPP5 | 0.611 |  |  |  |  |  |  |
|  | AAPP6 | 0.655 |  |  |  |  |  |  |
|  | AAPP7 | 0.648 |  |  |  |  |  |  |
|  | AAPP8 | 0.558 |  |  |  |  |  |  |
|  | AAPP9 | 0.642 |  |  |  |  |  |  |
|  | AAPP10 | 0.68 |  |  |  |  |  |  |
|  | AAPP11 | 0.585 |  |  |  |  |  |  |
| Privacy Concern | PC1 |  |  |  |  |  | 0.875 |  |
|  | PC2 |  |  |  |  |  | 0.688 |  |
|  | PC3 |  |  |  |  |  | 0.778 |  |
|  | PC4 |  |  |  |  |  | 0.973 |  |
